# Pier Luigi Luisi (1938–2025)

**DOI:** 10.3390/life16010112

**Published:** 2026-01-13

**Authors:** Alejandro Hochkoeppler, Mauro Giustini, Pasquale Stano, Richard M. Thomas, Peter Walde

**Affiliations:** 1 Department of Pharmacy and Biotechnology (FaBiT), University of Bologna, 40129 Bologna, Italy; 2 Department of Chemistry, Sapienza University of Rome, 00185 Rome, Italy; mauro.giustini@uniroma1.it; 3 Department of Biological and Environmental Sciences and Technologies (DiSTeBA), University of Salento, 73100 Lecce, Italy; pasquale.stano@unisalento.it; 4 Department of Materials, ETH Zürich, 8093 Zürich, Switzerland; rmt1@rmthomas.org (R.M.T.); peter.walde@mat.ethz.ch (P.W.)

Pier Luigi Luisi was an inspiring scientist who instilled originality in research and who had a propensity to tackle difficult and unusual problems. As he sometimes said, the most challenging ideas at least deserve to be tested using the left hand! To the sadness of all those who were fortunate to know him, Luisi died in Rome on 26 August 2025, aged 87. He will be particularly remembered for the way he led his research group, the members of which enjoyed maximum freedom and were motivated by him to collaborate and broaden their interests both within and beyond science.

During his post-doctoral studies, Luisi became interested in studying the mechanisms of enzymatic catalysis, particularly using dehydrogenases as model systems. He maintained this research interest after he moved, in 1970, to The Swiss Federal Institute of Technology (ETH), Zürich, initially in the position of Oberassistent. Remarkably, performing pioneering stopped-flow experiments, Luisi demonstrated the functional inequality of the subunits of equine liver alcohol dehydrogenase [1]. He broadened his interests to include the investigation of the catalytic action of enzymes in unconventional fluids such as water-in-oil microemulsions (reverse micelles), engaging his research group in this new opportunity for pure and applied enzymology [2].

In 1984 the ETH elected Luisi to the position of Professor of Macromolecular Chemistry. Developing his ideas and intrigued by the possible origins of life, Luisi decided to take an innovative approach. In particular, his search for auto-reproducing micelles and vesicles and their use as hosts for replicating macromolecules represents an important contribution to the understanding of the features underlying a minimal and primordial cell [3,4]. Through intensive discussions and a collaboration with Francisco Varela, Luisi introduced the term “chemical autopoiesis” as a guiding principle for a chemical system that becomes a living system. This new area of research became a primary focus for Luisi [5], who continued to challenge the construction of a semi-synthetic minimal cell after he moved to Rome in 2003. Moreover, Luisi expanded his interest beyond investigating just the origins of life [6] to the contemplation of its very meaning, resulting in the book “The Systems View of Life. A Unifying Vision” which he co-authored with Fritjof Capra [7].

Despite this wide area of activity, Luisi’s interest in proteins and enzymes never weakened, leading him to report a study in 2006 on the “never-born proteins”, i.e., representatives of the protein family coded for by amino acid sequences not found in nature but nevertheless capable of forming defined structures [8].


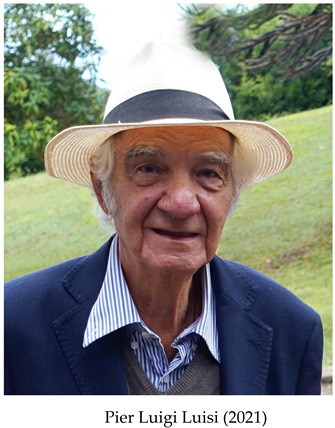


Pier Luigi Luisi was born on 23 May 1938, in Piombino, Tuscany (Italy) but spent most of his childhood on the island of Elba. He studied chemistry at the Scuola Normale Superiore di Pisa from where he graduated, cum laude, in 1963. He subsequently spent post-doctoral years in Leningrad (working with M. V. Volkenstein at the Institute of High-Molecular Compounds) and in Eugene (working with S. A. Bernhard at the University of Oregon, USA). He was twice married: firstly to Chris, with whom he had two sons, Peter and David, and secondly to Claudia. Following his retirement from the ETH as Professor Emeritus in 2003, he continued his research in the Università degli Studi Roma Tre, in Rome.

His contributions beyond pure science were many and varied. In 1984 he conceived of and instituted the “Cortona Week”, an interdisciplinary summer school in which scientists, artists and personalities from a wide variety of fields interact freely, exchange ideas and devise new approaches to different challenges. When (in 2017) the ETH finally withdrew support, it was felt that Luisi’s original concept was too important to lose and the project has been continued as ‘The Cortona Friends’, who meet and hold workshops annually. Luisi also wrote a number of novels and short stories, including a series, inspired by his book, “L’ombra dei fichi d’india. Storie elbane quasi tutte vere” [9] and dedicated to his beloved Elba. Quite a number of these books were illustrated by Hong Zhang, Luisi’s last partner with whom he spent most of his years in Rome. His memory will forever be cherished by all those who knew him.

On behalf of all co-workers who assisted Pier Luigi Luisi in his research at ETH Zürich and Università degli Studi di Roma Tre:

Alejandro Hochkoeppler, Mauro Giustini, Pasquale Stano, Richard M. Thomas, Peter Walde.

## Data Availability

Not applicable.
